# Elective Cesarean Section on Term Pregnancies Has a High Risk for Neonatal Respiratory Morbidity in Developed Countries: A Systematic Review and Meta-Analysis

**DOI:** 10.3389/fped.2020.00286

**Published:** 2020-06-25

**Authors:** Maleda Tefera, Nega Assefa, Bezatu Mengistie, Aklilu Abrham, Kedir Teji, Teshager Worku

**Affiliations:** ^1^School of Nursing and Midwifery, College of Health and Medical Sciences, Haramaya University, Harar, Ethiopia; ^2^Department of Environmental Health Sciences, College of Health and Medical Sciences, Haramaya University, Harar, Ethiopia

**Keywords:** respiratory morbidity, newborn, elective cesarean section, spontaneous vaginal delivery, term pregnancies

## Abstract

**Background:** Cesarean section (CS) is one of the most recurrently carried out surgical procedures in modern obstetrics. Worldwide, about 18.5 million CSs are conducted annually. Of this, 21–33% are performed in middle-and high-income countries. The effectiveness of the CS in preventing maternal and prenatal mortality and morbidity is medically justifiable. However, cesarean delivery without demanding obstetrical indications, by mere maternal request, may expose the child to several risks over benefits. Therefore, we aim to compare spontaneous vaginal delivery (vaginal delivery other than operative vaginal deliveries) and elective CS (CS before the onset of labor, but not including emergency CS) in decreasing the risk of neonatal respiratory morbidity.

**Objective:** To compare the risk of neonatal respiratory morbidity in ECS and spontaneous vaginal delivery.

**Methods:** A literature search was performed through visiting an electronic database (MEDLINE, PubMed, EMBASE, and CINAHL) and gray literature sources, including Google and Google Scholar, from January 2000 to May 2018. Original observational studies that reported the risk of neonatal respiratory morbidity in relation to mode of delivery conducted in the English language were identified and screened. Joanna Briggs Institute's quality assessment tool for observational studies was used to critically appraise the methodological quality of studies. Synthesis of individual studies was conducted using the Review Manager Software version 5.3 for Windows. Heterogeneity among studies was explored using the Cochran's *Q*-test and the *I*^2^ statistics. Pooled effect sizes in relative risk ratios with 95% confidence intervals were calculated. The flow of the study was prepared according to the Meta-analysis of Observational Studies in Epidemiology (MOOSE) checklist.

**Results:** Sixteen studies were reviewed. A total of 327,272 neonates born by vaginal delivery and 55,246 born by ECS were included in this study. The risk of neonatal respiratory morbidity was increased by 95% in neonates delivered by ECS (*RR* = 1.95; 95% CI: 1.40–2.73) as compared with neonates born by spontaneous vaginal delivery.

**Conclusion:** This study investigated the effect of mode of delivery on the respiratory morbidity without considering other risks and found that the ECS has a high risk of developing neonatal respiratory morbidities when compared to spontaneous vaginal delivery. So, we recommend discouraging unnecessary CS.

**registration**: CRD42018104905.

## Introduction

Cesarean section (CS) is one of the most recurrently carried out surgical procedures in modern obstetrics. About 18.5 million CSs are conducted yearly worldwide, and 21–33% of all CSs in excess are performed in middle and high-income countries (1, 2). The effectiveness of CS in preventing maternal and prenatal mortality and morbidity is justifiable medically, though there is no scientific confirmation that shows the benefit of cesarean delivery for the mother or for the newborn baby who does not require CS. Like other surgical procedures, CS has short and long-term risks, which may affect the reproductive health and physiological health of the woman and her child. These risks are higher in women with limited access to comprehensive obstetric care (3).

The United States vital statistics data have shown that the risk of neonatal mortality is increased by 1.5-fold after planned and unplanned CS compared to vaginal delivery, and the most common cause of neonatal mortality is respiratory morbidity (4). However, the incidence of birth trauma, meconium aspiration syndrome, and birth asphyxia is reduced by this mode of delivery as compared to vaginal delivery (5). Mostly respiratory morbidity occurs as a result of failure to clear fetal lung fluid (5). In recent times, studies have revealed that the incidence of respiratory morbidity [transient tachypnea neonatal (TTN), respiratory distress syndrome (RDS), or persistence pulmonary hypertension (PPH)] was 10% in neonates born by elective CS (ECS) at 37 weeks as compared to 2.8% among neonates born vaginally (5).

The risk of respiratory morbidity is significantly higher in neonates born with a CS before the onset of labor compared with a CS during labor (6), and the timing of the CS also affects the incidence of respiratory morbidity. The newborn who was born by ECS at 37 and 38 weeks' gestation had a higher risk of respiratory morbidity. As compared to intended vaginal delivery at 40 weeks, giving birth by ECS at 39 weeks' gestation still has an increased risk of respiratory morbidity (6–8).

The effect of the ECS on a newborn has remained controversial (9, 10). An understanding of the effect of the ECS on the neonatal respiratory outcome would help clinicians and policymakers to make the appropriate decision. In this review, we aim to evaluate the risk of respiratory morbidity in term singleton neonates delivered by ECS vs. spontaneous vaginal delivery (SVD), with ECS considered as an exposure variable, whereas vaginal delivery as the control group; the expected outcomes were neonatal respiratory morbidity (as primary outcome) and low Apgar score (as secondary outcome).

## Materials and Methods

### Searching Strategy

The whole search was conducted by three investigators [MT (PhD fellow), NA (PhD, associate professor), and TW (assistant professor)] who were trained in comprehensive searching strategies and comprehensive systematic review and meta-analysis, with the help of one senior librarian in our university. We contacted the authors for full information to abstract only articles.

### Sources of Studies and Searching Strategies

The literature search was conducted by visiting both electronic databases and gray literature sources. We used four databases to locate and retrieve the articles: CINAHAL, EMBASE, PUBMED, and MIDLINE. Google Scholar and Google were our gray literature sources. The searching term was as follows: “neonatal respiratory distress OR respiratory distress syndrome OR transient tachypnea of newborn OR persistence pulmonary hypertension AND cesarean section OR surgical procedures, operative OR vaginal birth, OR vaginal delivery obstetric surgical procedure AND full-term AND developed countries” The search was restricted to papers published in the English language and published from January 2000 to February 2018 (Additional Files 1).

The review flow was established based on the Meta-analysis of Observational Studies in Epidemiology (MOOSE) reporting guidelines (Table 1). It was based on the protocol registered by the International Prospective Register of Systematic Reviews (PROSPERO) of the University of York with the registration number of CRD42018104905.

**Table 1:** MOOSE checklist for meta-analyses of observational studies.

**Item no**.	**Recommendation**	**Reported on page no**.
**REPORTING OF BACKGROUND SHOULD INCLUDE**		
1	Problem definition	2
2	Hypothesis statement	2
3	Description of study outcome(s)	2–3
4	Type of exposure or intervention used	2–3
5	Type of study designs used	3
6	Study population	2–3
**REPORTING OF SEARCH STRATEGY SHOULD INCLUDE**		
7	Qualifications of searchers (e.g., librarians and investigators)	3
8	Search strategy, including time period included in the synthesis and keywords	3, Additional File 1
9	The effort to include all available studies, including contact with authors	3–4
10	Databases and registries searched	3
11	The search software used, name and version, including special features used (e.g., explosion)	4
12	Use of hand searching (e.g., reference lists of obtained articles)	3
13	List of citations located and those excluded, including justification	4, Table 2, Figure 1
14	Method of addressing articles published in languages other than English	–
15	Method of handling abstracts and unpublished studies	4
16	Description of any contact with authors	4
**REPORTING OF METHODS SHOULD INCLUDE**		
17	Description of relevance or appropriateness of studies assembled for assessing the hypothesis to be tested	4–5
18	The rationale for the selection and coding of data (discouraging, sound clinical principles, or convenience)	4
19	Documentation of how data were classified and coded (e.g., multiple raters, blinding and interrater reliability)	4–5
20	Assessment of confounding (e.g., comparability of cases and controls in studies where appropriate)	5
21	Assessment of study quality, including blinding of quality assessors, stratification, or regression on possible predictors of study results	5
22	Assessment of heterogeneity	5
23	Description of statistical methods (e.g., complete description of fixed or random effects models, justification of whether the chosen models account for predictors of study results, dose-response models, or cumulative meta-analysis) in sufficient detail to be replicated	5–6
24	Provision of appropriate tables and graphics	Tables 1, 2
**REPORTING OF RESULTS SHOULD INCLUDE**		
25	Graphic summarizing individual study estimates and the overall estimate	Figures 1–7
26	A table giving descriptive information for each study included	Table 2
27	Results of sensitivity testing (e.g., subgroup analysis)	Figure 3
28	Indication of statistical uncertainty of findings	6–7

**Table 2:** Description of the study characteristics for the included studies in the review.

**References and country**	**The aim of the study**	**Study design and participants**	**Data collection methods**	**Respiratory morbidity**	**Sample size**	**Total ESD**	**Event (RM)**	**Total VD**	**Event (RM)**
Breim et al. (11) Brazil	To compare the effects of the modes of delivery on the health of newborns	Cross-sectional retrospective design, including consecutively admitted patients for giving birth at the hospital, between January 1995 and December 1998	Perinatal medical chart review	RDS	304	249	44 (17.6)	59	11 (18.6)
Ceriani et al. (12) Italy	To compare the neonatal morbidity rate in low risk term infants delivered by vaginal or CD	Prospective cohort and analytical cohort study, infants ≥37 weeks born at the hospital between December 2004 and July 2006	Maternal chart review and observation	RDS, TTN	2,021	901	48 (5.3)	1,120	35 (3)
Dehdashtian et al. (13) Iran	To find out the incidence of respiratory distress in term neonates delivered by ESC and compare it with neonates delivered vaginally	Prospective epidemiological cross-sectional study, infants born by elective cesarean section or vaginal delivery at term	Chart review of mothers and their infants	TTN RDS, PPHN	1,000	500	27 (5.4)	500	8 (1.6)
Gyurkovits et al. (14) Hungary	To determine the incidence of RM according to gestational weeks, mode of delivery, and gender; and to assess whether the timing of delivery between 37 and 41 weeks gestation influences the neonatal respiratory outcome	A retrospective cohort was carried out on neonates born by elective cesarean section or vaginal delivery	Maternal and neonates chart review	TTN, RDS, MAS, PPHN	2,316	924	55 (6)	1,362	65 (5)
Heinzmann et al. (16) German	To specify neonatal outcomes following different modes of delivery	Retrospective cohort. All infants born at the Department of Gynaecology and Obstetrics, University of Freiburg, Germany, between 1st January 2004 and 31st December 2005	Chart review of the mothers	RDS	2,073	849	57 (6.7)	1,224	32 (2.6)
Karlstrom et al. (17) Sweden	To compare maternal complications and infant outcomes for women undergoing elective cesarean sections based on a maternal request and without recorded medical indication with those of women who underwent spontaneous onset of labor with the intention to have a vaginal birth.	A case-control study, women undergoing cesarean sections without medical indication and a control group of 13,774 women undergoing births through the spontaneous onset of labor	Medical Birth Register review	RDS	13,071	5,877	159 (2.7)	12,936	132 (1)
Herstad et al. (18) Norway	To examine the association between maternal age and adverse outcomes in low-risk primiparous women, and the risk of adverse outcomes by delivery modes	Cross-sectional study, low-risk primiparas with singleton, cephalic labors at ≥37 weeks during 1999–2009	Medical Birth Registry of Norway review	Respiratory compensation	7,372	5	373	6,999	82
Borgwardt et al. (10) Denmark	To investigate the association among ESC, spontaneous VD and neonatal RM in normal pregnancies expecting a normal uncomplicated birth	A retrospective cohort, neonates delivered by spontaneous vaginal delivery in cephalic presentation and ESC from 37 to 38 weeks gestation	Pt. administrative systems and clinical databases review	PPHN,TTN RDS	2,178	494	21 (4.2)	1,680	76 (4.5)
Liston et al. (19) Nova Scotia	To estimate the impact of cesarean delivery on the incidence of selected neonatal outcomes	Population-based, cohort analysis All deliveries occurring to a resident of Nova Scotia between 1 January 1988 and 31 December 2002, which resulted in a live-born singleton at term (>36 weeks) without any known major congenital anomalies	Perinatal Database review	RDS TTN	1,110,434	10,755	235 (2.2)	99,679	722 (0.7)
Liu et al. (20) China	To describe the risks and benefits of cesarean delivery on maternal request (CDMR)	Retrospective cohort study, singleton term nulliparous women with vertex presentation	Hospital electronic medical record review	RDS	49,166	16,333	90 (0.6)	32,833	148 (0.5)
Many et al. (8) Israel	To examine the rate of respiratory morbidity in neonates delivered by ECD at term, with a definite confirmation of gestational age	Prospective cohort women delivered sequentially by ECD at 38 1/7 weeks as exposure and consecutive women who had normal term vaginal delivery at 38 1/7 weeks	Maternal data record review and observation	RDS, TTN	588	277	5 (1.8)	311	0 (0)
Smith et al. (21) Scotland	To determine whether neonatal respiratory morbidity at term is associated with an increased risk of later asthma and whether this may explain previously described associations between cesarean delivery and asthma	Retrospective cohort study. All singleton births at term between 1992 and 1995 in 23 Scottish maternity hospitals	Scottish Morbidity Record review	RDS, TTN	158,010	10,240	453 (4.4)	147,770	1,366 (1)
Saddi et al. (22) Australia	To evaluate the association between gestational age at delivery and neonatal respiratory outcomes after elective cesarean delivery between 37 and 41 completed weeks	Prospectively cohort study. All singleton term infants (who completed 37–41 gestational weeks) who were born by ECS and SVD	Observation	RDS TTN		1,428	108(7.6)	10	1,304(0.8)
Thavarajah et al. (23) Australia	To investigate the relationship between the 5-min Apgar score categories (low, intermediate, and normal), mode of birth and neonatal outcomes	A retrospective cross-sectional (observational), all public, the term (_37 weeks), non-anomalous, singleton pregnancies	Hospital's electronic maternity database and maternal and fetal medicine and neonatology database review	RDS	28,100	4,411	213 (5)	23,689	488 (2)
Zanardo et al. (24) Italy	To establish whether the timing of delivery between 37 and 41 _weeks gestation influences the neonatal respiratory outcome in elective cesarean delivery, following uncomplicated pregnancy	The retrospective cohort, All pregnant women who were delivered by elective cesarean delivery at term during a 3-year period	Database comprising maternal and neonatal information drawn from the medical chart review	RDS	2,568	1,284	41 (3)	1,284	16 (1.2)
Wankaew et al. (9) Thailand	To evaluate the morbidities and mortality of neonates delivered by elective repeated cesarean section vs. normal vaginal delivery among women with uncomplicated term pregnancies	A retrospective cross-sectional study, all uncomplicated term pregnancies at Srinagarind Hospital	Maternal chart review	RDS, TTN	4,653	372	9 (2.4)	1,581	63 (4)

*ESD, elective caesarian section; MAS, meconium aspiration syndrome; PPHN, persistent pulmonary hypertension of the newborn; RDS, respiratory distress syndrome; RM, respiratory morbidity; TTN, transient tachypnea of the newborn; VD, vaginal delivery*.

### Study Selection

We include all observational studies published in English that compare the risk of neonatal respiratory morbidity in term singleton newborn infants delivered by ECS and those who were delivered by SVD. The participants of the studies were term singleton neonates born by SVD and ECS, without congenital malformation in developed countries (based on World Bank Economic Classification). This search included all published and unpublished observational (prospective cohort, cross-sectional, comparative cross-sectional, retrospective cohort, and case-control) studies done in developed countries on the effect of mode of delivery on neonatal respiratory morbidity conducted from January 1, 2000 to May 30, 2018 and written in the English language.

**Figure 1:**
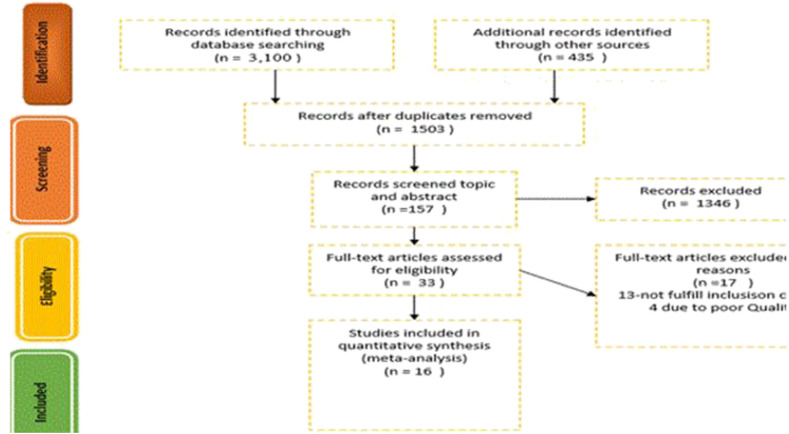
PRISMA flow chart.

We excluded the studies without a comparison group and compared ECS with emergency CS. Different modes of deliveries and studies that did not differentiate between ECS and emergency CS were also excluded. In addition, studies that focused on preterm and twin births were excluded from the review. Finally, 16 studies were identified; the details are presented in a PRISMA flowchart (Figure 1).

### Study Selection Procedure Screening

The identified studies from electronic and other relevant sources were exported to an EndNote citation manager and duplicate studies were removed. The four authors (MT, BM, AA, and KT) screened the studies based on the information contained in the topic and abstract independently. Based on the screening result, the studies that did not fulfill the inclusion criteria were excluded from the review. Then the full text of included and undecided studies was obtained for further screening.

To screen the final studies, the authors (MT, AA, and KT) independently reviewed the full text of included and undecided studies against eligibility criteria and critical appraisal; finally, 16 studies were selected. Critical appraisal was done by using JBI checklists for observational studies (Additional File 2).

### Data Extraction Process

All data were extracted using a structured data extraction template, a summary table prepared in Microsoft Word and Excel. The summary table encompassed the following: study author and year, study design, sample size, data collection method, and outcome of the study. Extraction was conducted by three authors (MT, NA, and BM). During the data extraction, neonates who were delivered by ECS were included in the exposed group and those who were delivered by SVD were included in the non-exposed (reference) group.

### Risk of Bias (Quality) Assessment

#### Assessment of Methodological Quality

All included studies were assessed for methodological validity by the authors independently using the JBI (Joanna Briggs Institute) checklists (Additional File 2). Special focus was given to the objective of the study for clearly identifying variables to be measured, identification of study inclusion and exclusion criteria, use of the probability sampling technique and preciseness of outcome interest measurement and appropriate statistical model, as well as identification and handling of sources of bias or confounding factors (Additional File 3).

#### Strategy for Data Synthesis

Synthesis of individual studies was conducted using the Cochrane community Review Manager Software (RevMan version 5.3 for windows). Summary statistics (pooled effect sizes) in relative risk ratios with 95% confidence intervals were calculated. The meta-analysis results were presented using a forest plot and summary table. Presence of statistical heterogeneity was tested by using the chi-squared test (Cochran's *Q*-test) and forest plot at a *P* ≤ 0.05. The level of heterogeneity among the studies was quantified using the *I*^2^ statistics, where substantial heterogeneity was assumed if the *I*^2^ value was ≥50%. During the analysis, ECS (CS before the onset of labor, but not including emergency CS) was considered as an exposure group whereas vaginal delivery (vaginal delivery other than operative vaginal deliveries) was considered as a control group; gestational age was categorized as an early term (37 and 38 weeks), late term (>40 weeks), and 39 weeks of gestation.

## Results

### Description of the Studies

We got 3,505 studies through searching the medical electronic database and other important sources. From those identified studies, 2,002 articles were removed due to duplication; the remaining 1,503 articles were screened by topic and abstract. Of these, 1,346 studies were excluded because the content presented in the title and the abstract did not match with our study. The remaining 157 studies with full text were reviewed for eligibility, and 124 studies were excluded due to an inconsistent study outcome and due to having different study populations compared with our study. The last 33 studies were critically appraised, and the studies that got a higher score were included in our study. Finally, 16 studies were included in this study (Figure 1). Of this, three retrospective cohorts, five prospective cohorts, seven cross-sectional studies, and one case-control study were analyzed. Almost all studies were adjusted for confounding variables, such as smoking, BMI, marital status, number of pregnancy, maternal age, ethnicity/race, sex of the infant, and anesthesia. But only two studies considered the effect of time of delivery on neonatal respiratory morbidity; the remaining 14 studies neither assessed the effect of timing nor controlled the gestational age as a confounding factor.

### Respiratory Morbidity and Mode of Delivery

Sixteen studies assessed the incidence of respiratory morbidity in relation to the mode of delivery. In almost all studies that have been reported, there is a significant relationship between the respiratory morbidity and the mode of delivery. The incidence of respiratory morbidities is two to three times more prevalent in neonates delivered by ECS.

**Figure 2:**
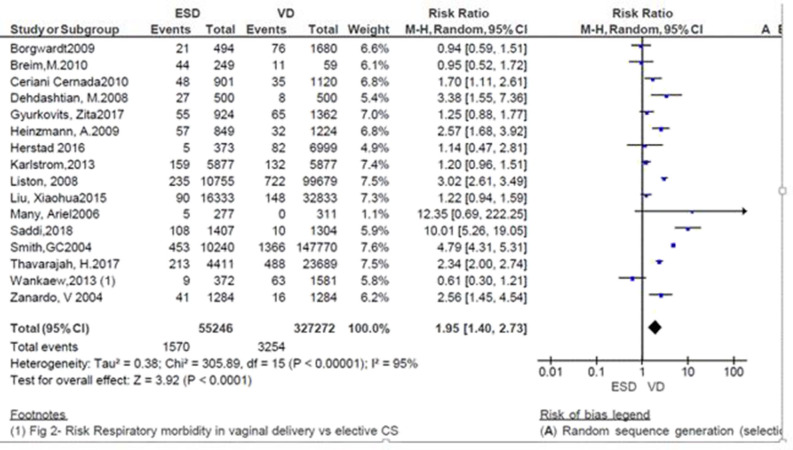
Risk of neonatal respiratory morbidity in spontaneous vaginal delivery vs. elective cesarean, 2019.

A total of 382,518 neonates were assessed in 16 studies; of those, 327,272 were neonates born by vaginal delivery and the rest (55,246) were by ECS. Except for three (9–11), all the studies showed that the incidence of respiratory morbidity was high in ECS (8, 12–14, 16–24). In particular, two studies showed that the risk of neonatal respiratory morbidity was 12.35 and 10 times more in neonates born by ECS (*RR* = 12.35; 95% CI: 0.66–222.25 and *RR* = 10.01; 95%CI: 5.26–19.05) (8, 22). On the other hand, three studies favor the ECS with non-significant association with respiratory morbidity (9–11). The pooled analysis showed that the independence of respiratory morbidity was increased by 95% in neonates delivered by ECS (*RR* = 1.95; 95% CI: 1.40–2.73; Figure 2).

### Subgroup Analysis and Publication Bias

**Figure 3:**
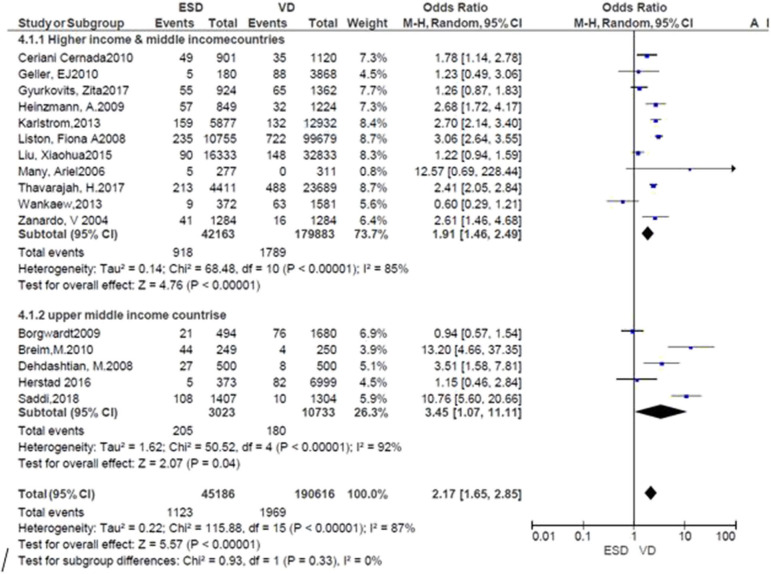
Subgroup analysis; risk of neonatal respiratory morbidity in spontaneous vaginal delivery vs. elective cesarean section at upper-middle income and higher-income countries, 2019.

Subgroup analysis was performed by the countries where the studies were conducted. The risk of neonatal respiratory morbidity was 1.91 times more in neonates delivered by ECS in high and middle-income countries (*RR* = 1.91; 95% CI: 1.46, 2.49), while it was 3.45 times more in the ECS group at upper-income countries. The result showed that the risk was high in upper-income countries. However, heterogeneity tests indicated that *I*^2^ = 85 and 92%, respectively. Sensitivity analysis was also done by removing the outlier and no significant difference was found (Figure 3). Publication bias was not detected in all studies and methods (Additional File 3).

### Effect of Time of Delivery on Neonatal Respiratory Morbidity

We got only one study in our study period that compared SVD and the ECS with the timing of birth, so we added one study done in 1995 (25).

### Risk of Neonatal Respiratory Morbidity at Early Term and Mode of Delivery

**Figure 4:**
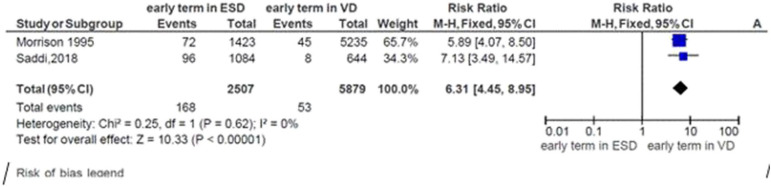
Risk of neonatal respiratory morbidity in early term and mode of delivery, 2019.

Two studies were included in the meta-analysis to assess the risk of neonatal respiratory morbidity in the early term in relation to the mode of delivery. The general risk ratio revealed that there was a significant association between early-term birth and the mode of delivery; the risk of respiratory morbidity was 6.3 times more likely to occur in early-term neonates delivered by elective cesarean section than early-term neonates born vaginally (*RR* = 5.53; 95% CI: 4.45, 8.595). The heterogeneity test indicated that *I*^2^ = 0%; hence, a fixed-effect model was assumed in the analysis (Figure 4).

### Risk of Neonatal Respiratory Morbidity at 39 Weeks of Gestation and Mode of Delivery

**Figure 5:**
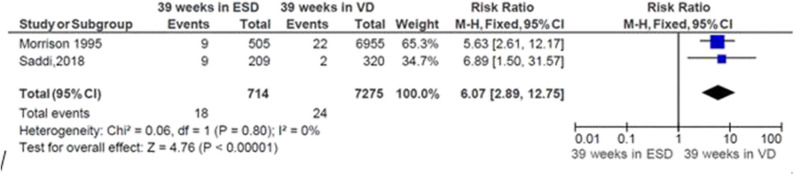
Risk of neonatal respiratory morbidity at 39 weeks of gestation and mode of delivery, 2019.

The pooled analysis showed that the risk of respiratory morbidity significantly increased by 507% in neonates delivered by ECS at 39 weeks of gestation relatively to neonates born by vaginal delivery at 39 weeks (*RR* = 6.07; CI 95%: 2.89, 12.75). The heterogeneity test indicated that *I*^2^ = 0%; hence, a fixed-effect model was assumed in the analysis (Figure 5).

### Risk of Neonatal Respiratory Morbidity at Late Term and Mode of Delivery

**Figure 6:**
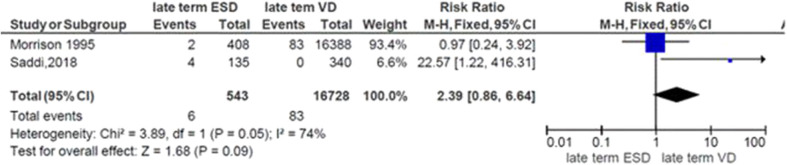
Risk of neonatal respiratory morbidity at late term and mode of delivery, 2019.

The general risk ratio revealed that there was a non-significant association between the late term of birth and the mode of delivery (*RR* = 2.39; 95% CI: 0.86, 5.64). The risk of respiratory morbidity was 2.4 times more in late-term neonates delivered by elective cesarean delivery (Figure 6).

### Apgar Score and Mode of Delivery

**Figure 7:**
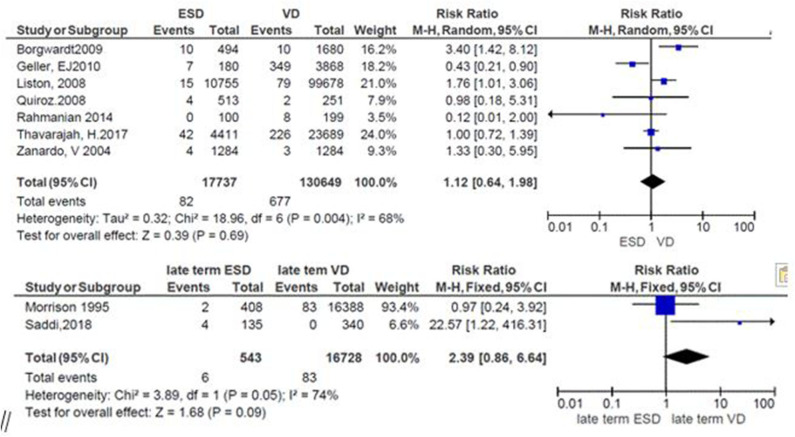
Low Apgar scores in relation to mode of delivery, 2019.

The relation between the mode of delivery and the 5-min low Apgar score was reported in seven studies. From these, three studies favor ECS (26–28) but only one of them showed significant association (*RR* = 0.43; 95%CI: 0.21, 0.90) (26); the same number of studies favors vaginal delivery (10, 19, 24), in which two studies showed significant association (10, 19) while one showed non-significant association (24). One study revealed that the risk of the Apgar score was the same in both groups (*RR* = 1.00; 95%CI: 0.72, 1.39) (23). The summary effect size demonstrates that the risk of the low Apgar score was almost similar in both modes of delivery but non-significantly higher in ECS (*RR* = 1.12; 95%CI: 0.64–1.18) (Figure 7).

## Discussion

Our systematic review and meta-analysis indicate that the risk of respiratory morbidity is high in ECS delivery and the most common respiratory problems were TTN and RDS in most eligible studies; less frequently, PPHN showed in some infants born by ECS.

Creating a smooth transition to air breathing is one of the major challenges a newborn faces after birth. This task is difficult because the fetal lung is full of fluid; to allow gas exchange, the fluid found in the fetal lung should be cleared rapidly. Failure to clear the fetal lung fluid results in respiratory morbidity, particularly in some infants delivered by ECS (15). The review demonstrates that the risk of neonatal respiratory morbidity is significantly increased with ECS as compared to vaginal delivery.

This result is supported by a multicenter study done at 11 hospitals in northeastern Italy that showed that the incidence of pulmonary disorder was high in ECS as compared to SVD (4.29 vs. 0.81%). According to this study, the pulmonary disorders considered are transient tachypnea of the newborn and respiratory distress syndrome (29). Similarly, a systematic review without meta-analysis analyzes nine studies comparing respiratory complications after ECS vs. vaginal delivery and revealed the range of risk, which was 20–70 per 10,000 with ECS and 10–20 per 10,000 with vaginal delivery birth (30).

Immediately after delivery, assessment should be done for newborn infants for early identification of newborn problems. One of the popular assessment tools is the Apgar score, which is a simple and effective method for assessing neonatal health in the immediate period after birth. The valid predictor of neonatal mortality, neurologic disability, and central auditory impairment is the low Apgar score at 5 min (31). We found that the delivery mode had non-significant association with 5-min Apgar scores. Even though the risk was better in vaginal delivery than ECS, a multicenter study of Maso and Monasta showed that the risk of a low Apgar score was higher in neonates delivered by CS as compared with vaginal delivery: 0.36 vs. 1.62% (29).

## Conclusion

This study investigated the effect of the mode of delivery on respiratory morbidity without considering other risks and found that neonates delivered via ECS have a high risk of developing neonatal respiratory morbidities when compared to those delivered via SVD. So, we recommend that an unnecessary CS should be discouraged by informing mothers. We also recommend other researchers to conduct RCTs regarding complications of respiratory morbidity and also its effect on families and society at large.

## Strengths and limitations

A major limitation of our meta-analysis was the number of studies (there were only two), which considered gestational age, and the heterogeneity of the study, which was due to the variation between studies in design, the characteristic of the study population, and medical and non-medical factors that caused the variation between studies. The study did not investigate other risks than respiratory morbidity. The major strength of our meta-analysis is that a comprehensive literature search was applied to include all studies in the area. Screening of the studies was based on our objective; to avoid duplication, a cautious exclusion of studies with overlapping populations was done. The final summary result was taken after critically appraising the studies.

## Data Availability Statement

All datasets generated for this study are included in the article/Supplementary Material.

## Author Contributions

MT, NA, BM, AA, KT, and TW conceived and designed the review. MT is the guarantor of the review and carried out the draft of the manuscript. MT, NA, and TW developed the search strings. MT, BM, AA, and KT screened and selected the studies. MT, NA, and BM carried out the analysis and interpretation, and rigorously reviewed the manuscript. All authors contributed to the article and approved the submitted version.

## Conflict of Interest

The authors declare that the research was conducted in the absence of any commercial or financial relationships that could be construed as a potential conflict of interest.

## Abbreviations

CI: confidence interval
RR: risk ratio
ESC: elective cesarean section
ESD: elective cesarean delivery
CS: cesarean section
VD: vaginal delivery
BMI: body mass index
RDS: respiratory distress syndrome
TTN: transient tachypnea neonatal
PPHN: persistence pulmonary hypertension
MAS: meconium aspiration syndrome
ABG: arterial blood gas.
