# Supplementary material for: Elective Cesarean Section on Term Pregnancies Has a High Risk for Neonatal Respiratory Morbidity in Developed Countries: A Systematic Review and Meta-Analysis
Source: Front Pediatr. 2020 Jun 25;8:286. doi: 10.3389/fped.2020.00286 (PMC7330011; doi:10.3389/fped.2020.00286)
Supplement: Supplementary file 1 [file Data_Sheet_1.docx]

Additional files 1: search strings of electronic databases

**PUBMED, EMBAS MIDLINE and CINAL search strategy**

((((((((respiratory[All Fields] AND distress[All Fields]) AND ("2000/01/01"[PDAT] : "2017/12/31"[PDAT]) AND "humans"[MeSH Terms] AND English[lang]) OR ("Respiratory Distress Syndrome, Newborn"[Mesh] AND ("2000/01/01"[PDAT] : "2017/12/31"[PDAT]) AND "humans"[MeSH Terms] AND English[lang])) OR ("Persistent Fetal Circulation Syndrome"[Mesh] AND ("2000/01/01"[PDAT] : "2017/12/31"[PDAT]) AND "humans"[MeSH Terms] AND English[lang])) OR (("transient tachypnea of the newborn"[MeSH Terms] OR ("transient"[All Fields] AND "tachypnea"[All Fields] AND "newborn"[All Fields]) OR "transient tachypnea of the newborn"[All Fields] OR ("transient"[All Fields] AND "tachypnea"[All Fields] AND "newborn"[All Fields]) OR "transient tachypnea of newborn"[All Fields]) AND ("2000/01/01"[PDAT] : "2017/12/31"[PDAT]) AND "humans"[MeSH Terms] AND English[lang])) AND ("Transient Tachypnea of the Newborn"[Mesh] AND ("2000/01/01"[PDAT] : "2017/12/31"[PDAT]) AND "humans"[MeSH Terms] AND English[lang])) OR (("caesarean section"[All Fields] OR "cesarean section"[MeSH Terms] OR ("cesarean"[All Fields] AND "section"[All Fields]) OR "cesarean section"[All Fields]) AND ("2000/01/01"[PDAT] : "2017/12/31"[PDAT]) AND "humans"[MeSH Terms] AND English[lang])) AND ("Cesarean Section"[Mesh] AND ("2000/01/01"[PDAT] : "2017/12/31"[PDAT]) AND "humans"[MeSH Terms] AND English[lang])) AND ("Term Birth"[Mesh] AND ("2000/01/01"[PDAT] : "2017/12/31"[PDAT]) AND "humans"[MeSH Terms] AND English[lang]) AND (("2000/01/01"[PDAT] : "2017/12/31"[PDAT]) AND "humans"[MeSH Terms])


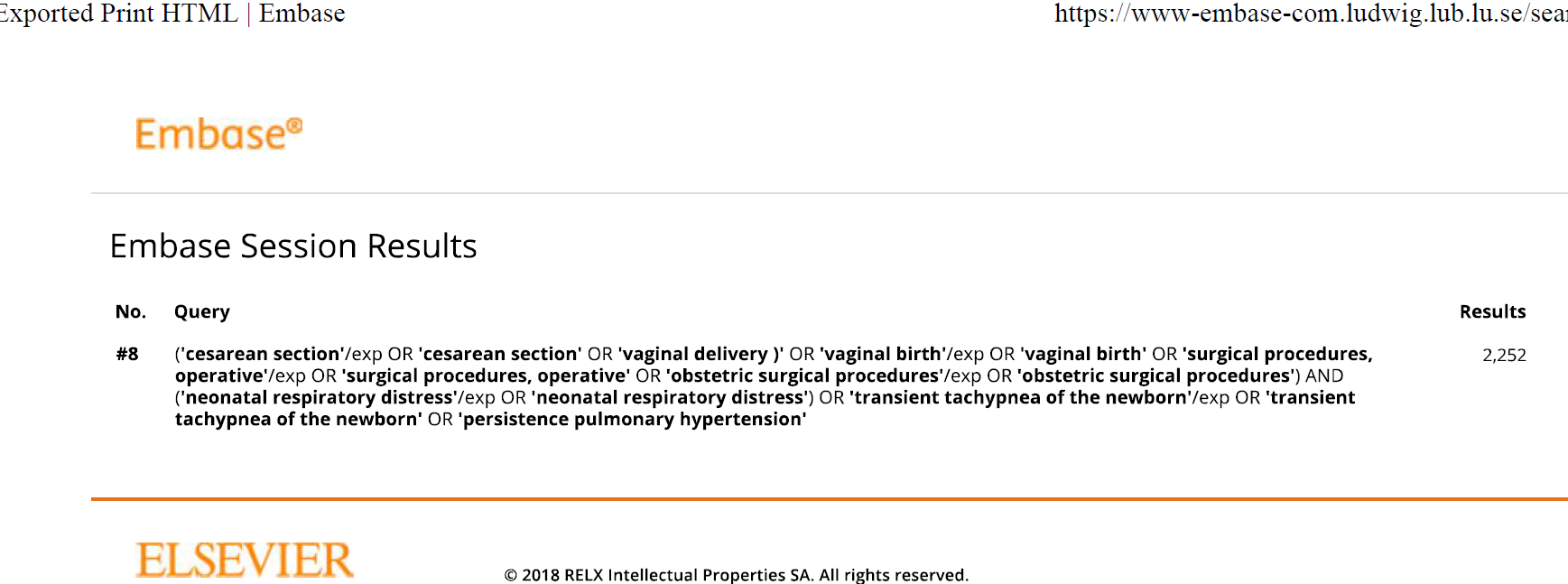


t
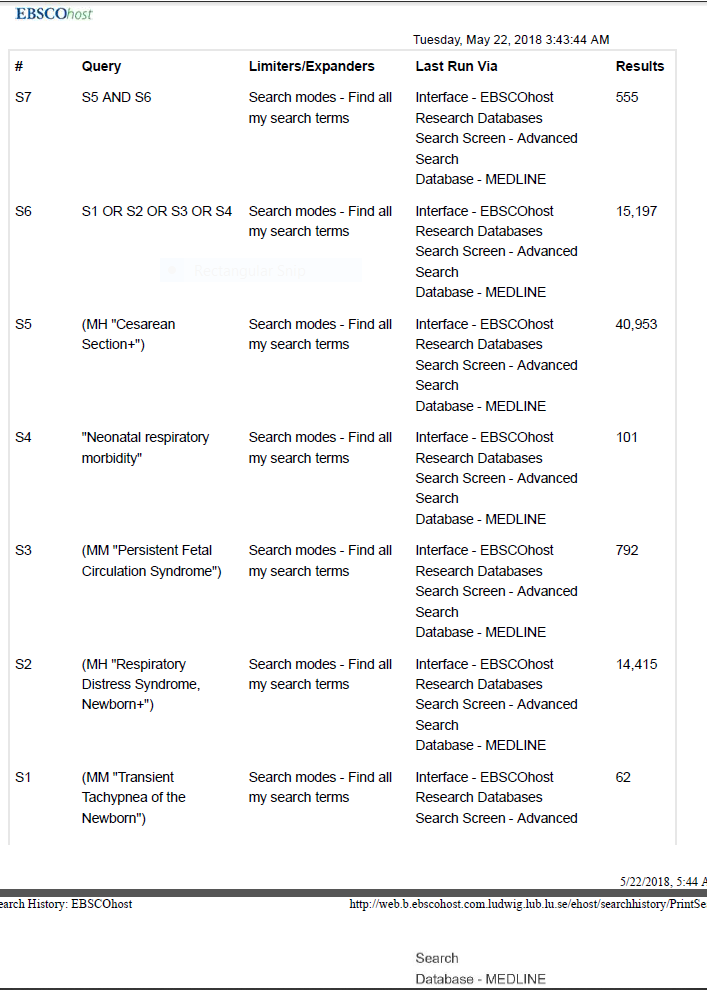


[Accessibility Information and Tips](javascript:openWideTip('http://support.ebsco.com.ludwig.lub.lu.se/help/?int=ehost&lang=en&feature_id=access&TOC_ID=Always&SI=0&BU=0&GU=1&PS=0&ver=&dbs=ccm')) Revised Date: 07/2015

**Print Search History**

|  | Thursday, May 17, 2018, 6:49:01 AM |
| --- | --- |

| **#** | **Query** | **Limiters/Expanders** | **Last Run Via** | **Results** |
| --- | --- | --- | --- | --- |
| S9 | S4 AND S6 | Limiters - Published Date: 20000101-20181231  Search modes - Find all my search terms | Interface - EBSCOhost Research Databases  Search Screen - Advanced Search  Database - CINAHL Complete | 124 |
| S8 | S4 AND S6 | Limiters - Published Date: -20181231  Search modes - Find all my search terms | Interface - EBSCOhost Research Databases  Search Screen - Advanced Search  Database - CINAHL Complete | 128 |
| S7 | S4 AND S6 | Search modes - Find all my search terms | Interface - EBSCOhost Research Databases  Search Screen - Advanced Search  Database - CINAHL Complete | 128 |
| S6 | S1 OR S2 OR S3 OR S5 | Search modes - Find all my search terms | Interface - EBSCOhost Research Databases  Search Screen - Advanced Search  Database - CINAHL Complete | 2,848 |
| S5 | "neonatal respiratory morbidity" | Limiters - Published Date: 20010101-20181231  Search modes - Find all my search terms | Interface - EBSCOhost Research Databases  Search Screen - Advanced Search  Database - CINAHL Complete | 63 |
| S4 | (MH "Cesarean Section+") OR (MM "Cesarean Section, Elective") | Search modes - Find all my search terms | Interface - EBSCOhost Research Databases  Search Screen - Advanced Search  Database - CINAHL Complete | 14,554 |
| S3 | (MM "Persistent Fetal Circulation Syndrome") | Search modes - Find all my search terms | Interface - EBSCOhost Research Databases  Search Screen - Advanced Search  Database - CINAHL Complete | 197 |
| S2 | "Transient Tachypnea of Newborn" | Search modes - Find all my search terms | Interface - EBSCOhost Research Databases  Search Screen - Advanced Search  Database - CINAHL Complete | 17 |
| S1 | (MH "Respiratory Distress Syndrome+") | Search modes - Find all my search terms | Interface - EBSCOhost Research Databases  Search Screen - Advanced Search  Database - CINAHL Complete | 2,620 |

Bottom of Form
