# Supplementary material for: Elective Cesarean Section on Term Pregnancies Has a High Risk for Neonatal Respiratory Morbidity in Developed Countries: A Systematic Review and Meta-Analysis
Source: Front Pediatr. 2020 Jun 25;8:286. doi: 10.3389/fped.2020.00286 (PMC7330011; doi:10.3389/fped.2020.00286)
Supplement: Supplementary file 2 [file Data_Sheet_2.docx]

| Author, year | Q1 | | | | Q2 | | | | Q3 | | | | | Q4 | | | | | Q5 | | | | Q6 | | | | Q7 | | | | Q8 | | | | Q9 | | | | Q10 | | | | Q11 | | | | Overall quality  result |
| --- | --- | --- | --- | --- | --- | --- | --- | --- | --- | --- | --- | --- | --- | --- | --- | --- | --- | --- | --- | --- | --- | --- | --- | --- | --- | --- | --- | --- | --- | --- | --- | --- | --- | --- | --- | --- | --- | --- | --- | --- | --- | --- | --- | --- | --- | --- | --- |
|  | Y | N | U | NA | Y | N | U | N | | Y | N | U | NA | | Y | N | U | NA | Y | N | U | NA | Y | N | U | NA | Y | N | U | NA | Y | N | U | NA | Y | N | U | NA | Y | N | U | NA | Y | N | U | NA |  |
| Ceriani et al 2010 | √ |  |  |  | √ |  |  |  | | √ |  |  |  | |  |  | √ |  |  |  | √ |  |  |  |  | √ | √ |  |  |  | √ |  |  |  | √ |  |  |  |  | √ |  |  | √ |  |  |  | 7/11(64%) |
| Gyurkovits, et al 2017 | √ |  |  |  | √ |  |  |  | | √ |  |  |  | | √ |  |  |  |  | √ |  |  |  |  |  | √ | √ |  |  |  | √ |  |  |  | √ |  |  |  |  | √ |  |  | √ |  |  |  | 8/11(73%) |
| Heinzmann, et al,2009 | √ |  |  |  | √ |  |  |  | | √ |  |  |  | | √ |  |  |  | √ |  |  |  |  |  |  | √ | √ |  |  |  | √ |  |  |  |  | √ |  |  |  | √ |  |  | √ |  |  |  | 7/1164%) |
| Line borg et al2009 | √ |  |  |  | √ |  |  |  | | √ |  |  |  | |  | √ |  |  |  | √ |  |  |  |  |  | √ | √ |  |  |  | √ |  |  |  | √ |  |  |  |  | √ |  |  | √ |  |  |  | 7/11(64%) |
| Liston, et al 2008 | √ |  |  |  | √ |  |  |  | | √ |  |  |  | |  | √ |  |  |  | √ |  |  |  |  |  | √ | √ |  |  |  | √ |  |  |  | √ |  |  |  |  | √ |  |  | √ |  |  |  | 7/11(64%) |
| Liu, et al 2015 | √ |  |  |  | √ |  |  |  | | √ |  |  |  | | √ |  |  |  | √ |  |  |  |  |  |  | √ | √ |  |  |  | √ |  |  |  | √ |  |  |  |  | √ |  |  | √ |  |  |  | 9/11(82%) |
| Many Ariel 2006 | √ |  |  |  | √ |  |  |  | | √ |  |  |  | | √ |  |  |  | √ |  |  |  |  |  |  | √ | √ |  |  |  | √ |  |  |  |  |  | √ |  |  | √ |  |  | √ |  |  |  | 8/11(73%) |
| Smith, et al 2004 | √ |  |  |  | √ |  |  |  | | √ |  |  |  | | √ |  |  |  | √ |  |  |  |  |  | √ |  | √ |  |  |  | √ |  |  |  | √ |  |  |  |  | √ |  |  | √ |  |  |  | 9/11(82%) |
| Saddi et al | √ |  |  |  | √ |  |  |  | | √ |  |  |  | | √ |  |  |  | √ |  |  |  |  |  |  | √ | √ |  |  |  | √ |  |  |  |  |  | √ |  |  | √ |  |  | √ |  |  |  | 8/11(73%) |
| Zanardo, et al 2004 Italy | √ |  |  |  | √ |  |  |  | | √ |  |  |  | |  | √ |  |  |  | √ |  |  |  |  |  | √ | √ |  |  |  | √ |  |  |  |  | √ | √ |  |  | √ |  |  | √ |  |  |  | 7/11(64%) |
|  |  |  |  |  |  |  |  |  | |  |  |  |  | |  |  |  |  |  |  |  |  |  |  |  |  |  |  |  |  |  |  |  |  |  |  |  |  |  |  |  |  |  |  |  |  |  |

**The critical appraisal for COHORT- studies**

****Y=yes, N=no, U=unclear, NA=not applicable, <60%=low, 60-80%=medium, >80%=high quality***

**The critical appraisal cross-sectional - studies**

****Y=yes, N=no, U=unclear, NA=not applicable, <60%=low, 60-80%=medium, >80%=high quality***

**The critical appraisal Case control studies**

****Y=yes, N=no, U=unclear, NA=not applicable, <60%=low, 60-80%=medium, >80%=high quality***

| Author, year | Q1 | | | | Q2 | | | | Q3 | | | | | Q4 | | | | | Q5 | | | | Q6 | | | | Q7 | | | | Q8 | | | | Q9 | | | | Q10 | | | | Overall quality  result |
| --- | --- | --- | --- | --- | --- | --- | --- | --- | --- | --- | --- | --- | --- | --- | --- | --- | --- | --- | --- | --- | --- | --- | --- | --- | --- | --- | --- | --- | --- | --- | --- | --- | --- | --- | --- | --- | --- | --- | --- | --- | --- | --- | --- |
|  | Y | N | U | NA | Y | N | U | N | | Y | N | U | NA | | Y | N | U | NA | Y | N | U | NA | Y | N | U | NA | Y | N | U | NA | Y | N | U | NA | Y | N | U | NA | Y | N | U | NA |  |
| Karlstrom, et al2013 | √ |  |  |  | √ |  |  |  | | √ |  |  |  | | √ |  |  |  | √ |  |  |  | √ |  |  |  | √ |  |  |  | √ |  |  |  |  | √ |  |  | √ |  |  |  | 9/10(90%) |

| Author, year | Q1 | | | | Q2 | | | | Q3 | | | | | Q4 | | | | | Q5 | | | | Q6 | | | | Q7 | | | | Q8 | | | | Overall quality  result |
| --- | --- | --- | --- | --- | --- | --- | --- | --- | --- | --- | --- | --- | --- | --- | --- | --- | --- | --- | --- | --- | --- | --- | --- | --- | --- | --- | --- | --- | --- | --- | --- | --- | --- | --- | --- |
|  | Y | N | U | NA | Y | N | U | N | | Y | N | U | NA | | Y | N | U | NA | Y | N | U | NA | Y | N | U | NA | Y | N | U | NA | Y | N | U | NA |  |
| Breim, et al. 2010 Brazil | √ |  |  |  | √ |  |  |  | | √ |  |  |  | | √ |  |  |  |  | √ |  |  |  | √ |  |  | √ |  |  |  | √ |  |  |  | 6/8(75%) |
| Dehdashtian, et al, | √ |  |  |  | √ |  |  |  | | √ |  |  |  | | √ |  |  |  |  | √ |  |  |  | √ |  |  | √ |  |  |  | √ |  |  |  | 7/8(88%) |
| Herstad et al 2016 | √ |  |  |  |  | √ |  |  | | √ |  |  |  | | √ |  |  |  | √ |  |  |  | √ |  |  |  | √ |  |  |  | √ |  |  |  | 6/8(75%) |
| Thavarajah et al, 2018 | √ |  |  |  | √ |  |  |  | | √ |  |  |  | | √ |  |  |  |  | √ |  |  |  | √ |  |  | √ |  |  |  | √ |  |  |  | 6/8(75%) |
| Wankaew et al n,2013 | √ |  |  |  | √ |  |  |  | | √ |  |  |  | | √ |  |  |  |  |  | √ |  |  |  | √ |  | √ |  |  |  | √ |  |  |  | 6/8(75%) |
|  | √ |  |  |  | √ |  |  |  | | √ |  |  |  | | √ |  |  |  |  |  | √ |  |  |  | √ |  | √ |  |  |  | √ |  |  |  | 6/8(75%) |
