# Supplementary figures and images for: Elective Cesarean Section on Term Pregnancies Has a High Risk for Neonatal Respiratory Morbidity in Developed Countries: A Systematic Review and Meta-Analysis
Source: Front Pediatr. 2020 Jun 25;8:286. doi: 10.3389/fped.2020.00286 (PMC7330011; doi:10.3389/fped.2020.00286)

**Additional file 3: Publication bias**


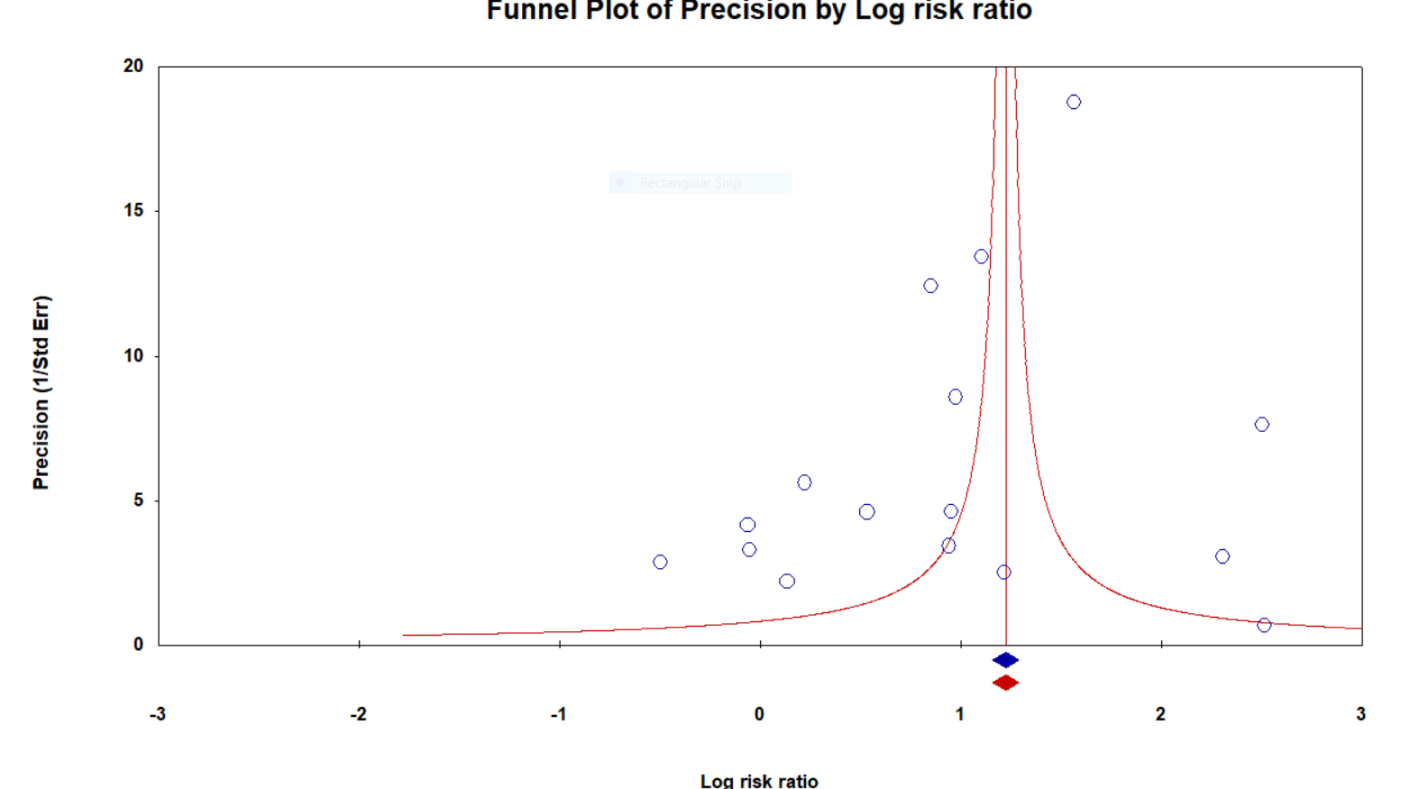


**Funnel plot**


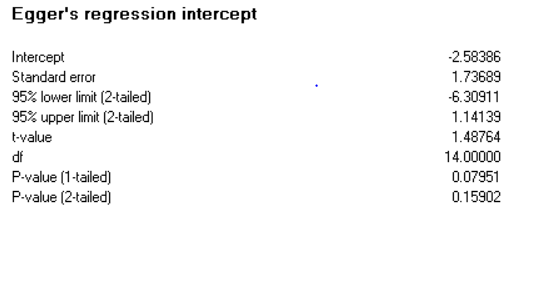


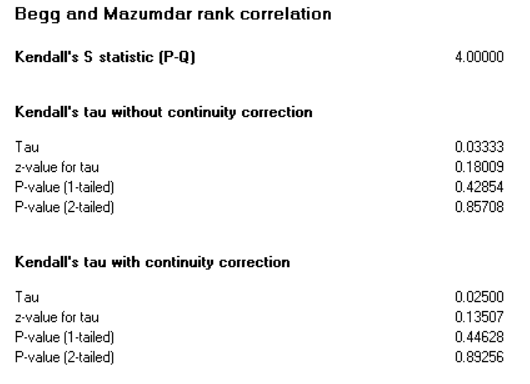

Supplement: Supplementary file 3 [file Data_Sheet_3.docx]
